# The polypill in the primary prevention of heart attacks and strokes: Overcoming barriers to implementation

**DOI:** 10.1177/09691413241235486

**Published:** 2024-03-15

**Authors:** Nicholas J Wald, Aroon D Hingorani, Stephen H Vale, Jonathan P Bestwick, Joan Morris

**Affiliations:** 1 Institute of Health Informatics, University College London, London, UK; 2 Population Health Research Institute, 4915 St Georges University of London, London, UK; 3 3Institute of Cardiovascular Science, University College London, London, UK; 4 Logical Medical Systems, London, UK; 5 Centre for Preventive Neurology, Wolfson Institute of Population Health, 4617 Queen Mary University of London, London, UK

**Keywords:** Screening, NHS Health Check, polypill, heart attacks, strokes

## Abstract

This commentary, linked to our paper in the same issue of the *Journal of Medical Screening*, discusses the reluctance to consider and adopt the polypill in the primary prevention of heart attacks and strokes, access to the polypill as a public health service, the formulation of the polypill in current use, its prescription as an unlicensed medicine, and what can be done to facilitate the adoption of the polypill approach as a routine public health service.

## Introduction

There is widespread recognition that more needs to be done in the primary prevention of heart attacks and strokes, which remain a leading cause of premature death and disability throughout the world. The polypill approach to primary prevention would have a major impact. Despite the polypill approach being set out and quantified in detail over 20 years ago, positive clinical trial results, and the evidence submitted to the World Health Organisation resulted in the polypill being added to its list of essential medicines in 2023^1^ (all references cited in our linked paper^2^), no large-scale polypill primary cardiovascular disease prevention programme has been implemented.

## Reluctance to adopt the polypill approach

There has been concern that the polypill approach has not been adopted despite its benefits.^3–5^ We here set out eight reasons that may account for this.

First, the magnitude of the benefit may not have been widely recognised. The flowchart analysis in the accompanying paper sets out and clarifies the practical outcome benefits of the polypill approach compared to the NHS Health Check Programme in England in a way that has not previously been done. Importantly it reveals the shortcomings of the NHS Health Check programme in preventing heart attacks and strokes.

Second, there may be concern that people who would never have a heart attack or stroke will be ‘exposed’ to medication. This also occurs with the multiple risk factor approach incorporated in the NHS Health Check, as we show in the accompanying paper, but it is not a justified concern because of the low cost and well-established safety of statins and blood pressure lowering drugs given in a low dose combination.

Third, the reluctance may reflect a conservative attitude to the use of medicines for disease prevention despite acceptance of the same medicines for the treatment of patients with the same disease. Concerns over ‘medicalisation’ are misplaced; the Polypill Prevention Programme has the opposite effect, in that it prevents a person from becoming a patient in the same way that people take anti-malarial medication to prevent contracting malaria. The issue is discussed in former *British Medical Journal* Editor Richard Smith's blog.^6^ In contrast, the NHS Health Check Programme makes a patient out of everyone selected for a statin.

Fourth, there is an unjustified perception that more scientific research is needed on the polypill approach. There is already sufficient evidence that a well-formulated polypill is safe and greatly reduces blood pressure and low-density lipoprotein (LDL) cholesterol which in turn prevents heart attacks and strokes.^7^ However unjustified the perception, there is a need to engage with, and persuade, policy makers of the advantages of the polypill approach.

Fifth, clinical guidelines have become increasingly focused on multifactorial risk prediction models and the addition of new predictors. This focus on prediction rather than prevention has added complexity, with recent updates to existing guidelines recommending incremental changes to existing models instead of taking a fresh look at the whole approach to the prevention of cardiovascular disease.

Sixth, and relatedly, professionals tend to overlook the fact that age is the most powerful predictor of cardiovascular disease, with little discrimination added by the addition of information from causal risk factors such as blood pressure and LDL cholesterol, or from ‘novel‘ biomarkers.

Seventh, there is little financial reward to the pharmaceutical industry in conducting expensive trials to obtain a primary prevention licence for a polypill which consists of generic medicines some at low non-standard doses. The use of a polypill as a ‘special’ medication is not attractive to the pharmaceutical industry because, although ‘specials’ are regulated, they are not licensed and therefore cannot be marketed.

Eighth, regulations to obtain a product license lack flexibility, tend to rely exclusively on randomised trials performed in the population of intended use (a major challenge in primary prevention) and are not proportionate to the evidence on benefits and hazards that is already available from trials and cohort studies that were not performed in the population of intended use.

None of these issues should be a barrier to adopting age-only screening and the use of a polypill as part of a low-cost, effective and safe public health service.

## Access to the polypill as a public health service

The polypill approach is a public health primary cardiovascular disease prevention programme that is based on age alone and the use of an optimally formulated polypill. It has advantages over a multiple risk factor screening approach that focuses on disease prediction and leaves preventive treatment to doctors. The polypill approach is simpler, avoids medical consultation, saves practitioner time, and avoids the need to carry out periodic blood pressure measurements and blood tests. Medical practices could identify people once they have reached age 50 and offer them, by email, post or text message, a polypill. This would be a standard formulation very much like a vaccine and could be dispensed by local pharmacies. The ethos would be to take the medication to avoid becoming a patient, not because one had become one, recognising that if a first heart attack or stroke is prevented there is no second one to prevent. The polypill approach as a public health service rests on a simple but significant change in the medical perspective. From age 50 it is better to lower blood pressure and LDL cholesterol in all and measure it in some, rather than measure them in all and lower it in some as is current practice. Put simply, prevention is better than measurement. The epidemiological and clinical trial evidence also indicates that there is no need to measure blood pressure or cholesterol prior to initiating preventive treatment because there is no threshold level below which further reduction will not confer some benefit; the higher the starting level the greater the benefit.^8,9^

Asking a few simple online questions can identify contra-indications to receiving a polypill as is done in the Polypill Prevention Programme (www.polypill.com). Monitoring is carried out online using symptom questions asked when a repeat prescription is requested. A doctor (or other health professional) reviews the answers to a few questions and, if appropriate, issues a repeat prescription which is transmitted electronically to a pharmacy that dispenses the polypill and couriers it to the polypill participant. The current private polypill service could be scaled up and offered by the NHS and other healthcare providers throughout the world. The service could be audited to determine what proportion of people offered the polypill take it up. There would be little financial waste because the cost would be approximately directly proportional to the uptake. Sample surveys of individuals could be conducted to determine the LDL cholesterol and blood pressure levels in participants in the prevention programme and in non-participants.

## Formulation of the polypill in current use

An appropriate and validated preventive medication for cardiovascular disease comprises a polypill that combines a statin and three low-dose blood pressure-reducing agents from different classes, preferably formulated in a single capsule or tablet.^10^ Such a multiple low-dose approach minimises any side effects of the blood pressure-lowering medicines. As a matter of routine practice, if a statin is indicated, so should a blood pressure lowering medication and vice versa because the aim is disease prevention, not to ‘normalise’ blood pressure or LDL cholesterol. ‘Normal’ levels in the population are typically too high and the benefit of lowering levels extends across the range of values in the population, including values that are below average. The benefit of the NHS Health Check programme is constrained by not routinely prescribing blood pressure lowering medications in people selected for prescription of a statin. Only about an average of 4 years of life are gained without a heart attack or stroke using a statin alone among the one in three people who, in the absence of preventive action, would be affected by a heart attack or stroke over their lifetime, instead of about 8 years if both a statin and blood pressure lowering medications are prescribed, calculated using standard life-table methods.^2^^,11,12^ A pill that contains both a statin and three low-dose blood pressure medications ensures that all are used together.^13^ The formulation of the polypill used in the UK Polypill Prevention Programme (www.polypill.com) is: rosuvastatin (10 mg), hydrochlorothiazide (12.5 mg), amlodipine (2.5 mg) and losartan (25 mg).

Aspirin could be considered as an additional component in a polypill, with a modest added benefit in preventing cardiovascular disease and a reduction in the incidence of colon cancer and possibly other cancer.^14–16^ Although aspirin increases the risk of a gastric bleed this is rarely fatal, and the balance is in favour of aspirin being used with a polypill. It is less certain whether folic acid, a suggested option in the original polypill proposal, should also be taken. Randomised trial evidence suggests that folic acid has a benefit in the prevention of stroke^17^ but has not shown a benefit in the secondary prevention of heart attack.^18^ This may be because the routine use of low-dose aspirin in the secondary prevention of cardiovascular disease has an anti-platelet effect that is not enhanced by the addition of folic acid.^19^ If aspirin is not used there is probably an additional benefit in taking folic acid; 0.8 mg per day is an appropriate regime daily dose because it has a maximal effect in lowering serum homocysteine.^20^

## The polypill as an unlicensed medicine

The polypill prescribed in the Polypill Prevention Programme is an unlicensed medicine but this is not a barrier; indeed it has several advantages. The UK regulations covering unlicensed medicines are particularly suitable for formulations that come under the general term ‘polypill’ and this is likely to be the same in other countries. An unlicensed medicine cannot be specifically promoted; it can however be formulated and prescribed to fit a special need that is not covered by a licensed formulation. The cost of producing and conducting a randomised trial of a polypill with particular components at specific doses to show that each component exerts an independent effect in preventing or treating a particular disorder, or set of disorders, is prohibitive. There is no commercial justification for such a trial when individual licensed preparations can be prescribed separately, with some tablets split to achieve the desired dose. The difficulty is magnified enormously when the aim is the primary prevention of heart attacks and strokes because of the much greater number of people needed in a trial to achieve statistical power. There is also the advantage that the composition and dose of a formulation can be readily varied; if it were licensed it would require fresh trials. Given that there is no need to advertise specific polypill formulations, there is no need to license them. There is no reason why the current service on polypill.com should not be done on a large scale so it is available on a population-wide basis with the cost savings arising from the economies of scale. The large-scale use of an unlicensed polypill is entirely possible but may face challenges. Regulators would need to see this as a positive public health opportunity and public education would be needed to explain that ‘unlicensed’ does not mean unregulated or substandard. Meeting these challenges should not be a reason for the NHS and other health services to further delay the implementation of the polypill approach as a national preventive service.

## Moving to the polypill approach

In May 2023, NICE issued updated guidance that recommends the use of statins in those who have a 10-year QRISK3 score of 10% or more, but not to rule out statin treatment ‘because the person's ten-year QRISK3 score is less than 10% if they have an informed preference for taking a statin or there is a concern that risk may be underestimated’.^21^ The guidance, however, is silent on concurrent blood pressure reduction, which has as large an effect on stroke prevention as statins have on preventing heart attacks. Notwithstanding this omission, the risk thresholds for preventive medication have fallen over time, from 20% to 10% 10-year risk – effectively moving towards a simple age-alone polypill-based programme. In this context, it is significant that in April 2023 the Hewitt Review^22^ commissioned by the UK Government endorsed the view that the primary prevention of cardiovascular disease was a national public health priority. A milestone in the acceptance of the polypill was passed in July 2023 when the World Health Organisation listed a polypill for the primary and secondary prevention of cardiovascular disease on its list of essential medicines.^23^

Despite these initiatives and the evidence of benefit, the polypill approach has not been adopted as general policy in over two decades, since it was first described. This alone indicates that more needs to be done before policymakers are willing to implement change. To this end, it would be appropriate to conduct a large-scale demonstration project of implementing a Polypill Prevention Programme and which compared it with current practice. A simple low-cost cluster randomisation design could be adopted, with integrated care systems being allocated to the polypill approach or usual practice, without the need for individual consent. Clinical outcomes could be sought using record linkage to electronic medical data. Health records could also be used to retrieve blood pressure and LDL cholesterol measurements and determine the differences between the polypill and current practice. This implementation project would provide direct evidence that could not be reasonably ignored.
